# Small Extracellular Vesicles Derived from Human Umbilical Cord Mesenchymal Stem Cells Enhanced Proangiogenic Potential of Cardiac Fibroblasts via Angiopoietin-Like 4

**DOI:** 10.1155/2022/3229289

**Published:** 2022-02-01

**Authors:** Jiejie Li, Xin Xu, Suyan Fei, Ren Wang, Hua Wang, Wei Zhu, Yuanyuan Zhao

**Affiliations:** ^1^School of Medicine, Jiangsu University, Zhenjiang, Jiangsu, China; ^2^The Affiliated Hospital of Jiangsu University, Zhenjiang, Jiangsu, China

## Abstract

**Methods and Results:**

We isolated primary CFs from Sprague-Dawley rats (1–3 days old) and treated them with lipopolysaccharide (LPS) and LPS+sEVs. RNA sequencing analysis revealed that angiopoietin-like 4 (Angptl4) was increased in the LPS+sEVs group more than in the LPS group. After inhibition of Angptl4 expression in sEVs and CFs, cell proliferation, Transwell migration, and tube formation assays were used to detect the angiogenic activity of human umbilical vein endothelial cells. *β*-Catenin expression in CFs was detected by western blotting. The *β*-catenin inhibitor ICG001 was used to examine whether *β*-catenin was involved in the proangiogenic potential of CFs promoted by sEVs. sEVs enhanced the proangiogenic potential of CFs under inflammatory conditions, which was associated with *β*-catenin signaling. The proangiogenic potential of CFs was decreased when Angptl4 was knocked down in CFs and in hucMSCs.

**Conclusions:**

The sEVs regulated CFs to promote angiogenesis via Angptl4 in an inflammatory environment. This may provide a research basis for treating myocardial injury with sEVs.

## 1. Introduction

Acute myocardial injury has a high mortality rate. The repair mechanisms after myocardial injury include inflammation suppression, enhanced angiogenesis, reduced fibrosis, and remodeling. Angiogenesis and restoration of blood supply are crucial for inflammation clearance and injury repair [1]. Therefore, promoting angiogenesis is considered a good choice for repair of the myocardial injury. Cardiac fibroblasts (CFs) are major noncardiomyocytes and play an important role in remodeling of damaged tissues [2]. The proangiogenic potential of CFs has also been reported. Saraswati et al. isolated two different fibroblast subtypes from mouse hearts after myocardial infarction and demonstrated that FSP1+ fibroblast had a proangiogenic role [3]. The role of CFs in cardiac injury repair can shift from inflammation to angiogenesis [4, 5]. It has been proven that mesenchymal stem cells (MSCs) or exosomes promote the repair of tissue injury and angiogenesis [6, 7]. Li et al. showed that stem cell-derived small extracellular vesicles (sEVs) promoted cardiac angiogenesis by delivering miR-486-5p, which is related to fibroblastic matrix metalloprotein 19 [8]. Exosomes derived from human endothelial progenitor cells increase proliferation and angiogenesis of CFs [9]. Our previous studies have demonstrated that exosomes derived from human umbilical cord MSCs (hucMSC-exs) had cardioprotective effects [10]. The hucMSC-exs also promote fibroblast-to-myofibroblast differentiation in the inflammatory stage and have cardioprotective effects [11]. However, whether sEVs can regulate CFs to promote angiogenesis in an inflammatory environment requires further research.

In this study, we isolated primary CFs from Sprague-Dawley (SD) rats (1–3 days old) and demonstrated that sEVs enhanced proangiogenic potential of CFs. The volcano map of differentially expressed genes (DEGs) and enrichment analysis of angiogenesis-related genes after RNA sequencing (RNA-seq) analyses of CFs showed that *Angptl4* might be a noteworthy gene. The angiopoietin-like 4 (Angptl4) is a member of the angiogenin-like protein family and is a secreted glycoprotein [12]. Therefore, we investigated the proangiogenic potential of Angptl4 in CFs in an inflammatory environment.

Several studies have demonstrated that the Wnt/*β*-catenin signaling pathway participated in regulating angiogenesis [13]. Angptl4 is also associated with *β*-catenin [14]. In this study, the role of *β*-catenin in the activity of CFs enhanced by sEVs was also investigated.

## 2. Materials and Methods

### 2.1. Animals

All animals' experiments were performed in accordance with the Guide for the Care and Use of Laboratory Animals and were approved by the Animal Experiment Center of Jiangsu University in Zhenjiang, Jiangsu Province, China. SD rats (1–3 days old) were used in this study.

### 2.2. Cell Culture

hucMSCs were isolated and cultured as described previously [15]. The umbilical cords were from the Affiliated Hospital of Jiangsu University (Jiangsu, China), and all providers gave informed consent. hucMSCs were cultured in Minimal Essential Medium Alpha (*α*-MEM; Gibco, Grand Island, NY, USA) with 10% fetal bovine serum (FBS; Biological Industries, Beit HaEmek, Israel) at 37°C in 5% CO_2_. The primary CFs were isolated from the hearts of 1–3-day-old SD rats according to an established method [16]. Human umbilical vein endothelial cells (HUVECs) were obtained from the American Type Culture Collection (Manassas, VA, USA) and cultured in high-glucose Dulbecco's Modified Eagle's Medium (H-DMEM; Gibco) with 10% FBS at 37°C in 5% CO_2_.

### 2.3. The sEV Extraction

When the hucMSCs reached 80% confluence in complete *α*-MEM, complete medium was replaced with *α*-MEM supplemented with 10% sEVs-depleted FBS. After 48 h, the supernatant was collected and centrifuged at 300 × *g* for 10 min, 2,000 × *g* for 20 min, and 10,000 × *g* for 30 min to remove debris. The supernatant was concentrated with 100 kDa molecular weight cut-off (MWCO) ultrafiltration centrifuge tubes (Millipore, Billerica, MA, USA). The concentrated supernatant was centrifuged at 120,000 × *g* for 70 min. The sedimentation at the bottom was resuspended in PBS. The mixture was centrifuged again at 120,000 × *g* for 70 min, and the sediment that contained the sEVs was resuspended in PBS and stored at -80°C.

### 2.4. Nanoparticle Tracking Analysis (NTA)

The isolated sEVs were diluted with PBS. The concentration and size distribution of sEVs were evaluated by using a Malvern Panalytical nanosight nanoparticle analyzer (Malvern, UK). The data were analyzed by using ZetaView version 8.05.12 SP1 software.

### 2.5. Electron Microscopy

The sEVs were mixed and 20 *μ*L was added to the sample loading copper mesh with a diameter of 2 mm, and an excess sample was removed. The copper mesh was inverted in a 2% phosphotungstic acid drop for 5 min at room temperature for negative staining. After drying, images were obtained with a transmission electron microscope (JEOL, Tokyo, Japan).

### 2.6. Flow Cytometry

When the density of hucMSCs reached 90%, cells were collected and incubated with antibodies against CD34, CD19, CD45, CD90, CD29, and CD105 (eBioscience, San Diego, USA) for 30 min at 4°C, washed three times, and resuspended in 300 *μ*L PBS. The data were immediately collected and analyzed with a flow cytometer (Becton Dickinson, Billerica, USA).

### 2.7. Immunofluorescence Analyses

When the density of CFs reached 70–80%, the supernatant was removed and the cells were washed three times with PBS. The cells were fixed with 4% paraformaldehyde at room temperature for 20 min and permeabilized with 0.1% Triton X-100 for 15 min. The cells were blocked with 5% bovine serum albumin (BSA) for 30 min at 37°C and incubated with primary antibodies against *α*-smooth muscle actin (*α*-SMA) (1 : 200; Boster, Birmingham, USA), vimentin (1 : 100; Cell Signaling Technology, Danvers, USA), collagen I (1 : 100; Boster), periostin (1 : 100; Proteintech, Rosemont, USA), CD31 (1 : 50; Arigo, Taiwan), and Angptl4 (1 : 100; Cell Signaling Technology) overnight at 4°C. The next day, the fluorescence-conjugated secondary antibodies (1 : 1,000; Cell Signaling Technology) were incubated with cells for 1 h at 37°C. The nuclei were counterstained with Hoechst 33342 (Sigma-Aldrich, Saint Louis, USA), and the images were viewed under a fluorescence microscope (OLYMPUS DP73, Tokyo, Japan).

### 2.8. Western Blotting

The proteins were isolated with RIPA lysis buffer containing protease and phosphatase inhibitors, PMSF, separated by 10–15% SDS-PAGE, and transferred to a PVDF membrane (Millipore, USA). Next, the PVDF membranes were blocked with 5% milk for 1 h and incubated overnight at 4°C with primary antibodies against CD9 (1 : 1,000; Cell Signaling Technology), TSG101 (1 : 1,000; Boster), CD81 (1 : 800; Proteintech), calnexin (1 : 1,000; Boster), *α*-SMA (1 : 1,000; Boster), vimentin (1 : 1,000; Cell Signaling Technology), collagen I (1 : 1,000; Boster), periostin (1 : 1,000; Proteintech), CD31 (1 : 1,000; Arigo), Angptl4 (1 : 800; Cell Signaling Technology), cyclin D1 (1 : 800; Cell Signaling Technology), *β*-catenin (1 : 800; Cell Signaling Technology), and GAPDH (1 : 2,000; Cell Signaling Technology). The next day, the secondary antibodies (1 : 3,000; Cell Signaling Technology) were incubated with the membranes for 1 h at 37°C. The bands were visualized by enhanced chemiluminescent image analysis.

### 2.9. RNA-seq Analyses

The primary CFs were treated with lipopolysaccharide (LPS) (100 ng/mL) and LPS+sEVs (200 *μ*g/mL) for 24 h. Total RNA was extracted and analyzed. All RNA sequencing data were obtained from BGI-Shenzhen (Shenzhen, China). All *P* values were corrected for statistical significance using the false detection rate (FDR) for multiple tests. Fold changes (absolute log2) ≥ 1 and FDR adjusted to *P* < 0.05 were considered statistically significant.

### 2.10. Angptl4 siRNA Transfection

Angptl4 siRNAs were constructed by RIBOBIO (China): siRNA1: 5′-GCAGAGTTTACAGACTCAA-3′, siRNA2: 5′-GCAGCCATTCCAATCTAAA-3′, and siRNA3: 5′-GGACAATACTTCCACTCTA-3′. When the density of cells reached 50–70%, the siRNAs or negative control (NC) RNAs were transfected into cells using Lipofectamine 2000 (Invitrogen, Carlsbad, NM, USA). After 6 h, the medium was replaced with fresh medium, and the cells were cultured for a further 48 h.

### 2.11. Reverse Transcription-Polymerase Chain Reaction (RT-PCR)

Total RNA was extracted with the TRIzol reagent (Invitrogen). The cDNA was obtained using RT-PCR kits (CWBIO, Cambridge, MA, USA). The PCR program was conducted with UltraSYBR mix (CWBIO) on a StepOnePlus Real-Time PCR System (Applied Biosystems, Foster City, CA, USA). The mRNA expression was normalized to *β*-actin. All the sequences of PCR primers are listed in Table 1.

### 2.12. Supernatant Preparation

The primary CFs were treated with PBS (control), LPS (100 ng/mL), LPS+sEVs (200 *μ*g/mL), or LPS+sEVs (200 *μ*g/mL)+ICG001(10 *μ*M) for 24 h. To further study the role of Angptl4, the primary CFs were transfected with NC-siRNA (NC-CF) or Angptl4-siRNA (si-CF) for 48 h and treated with LPS+sEVs, LPS+NC-siRNA-sEVs (LPS+NC-sEVs), and LPS+Angptl4-siRNA-sEVs (LPS+si-sEVs) for 24 h. The pretreated CFs were washed three times with PBS and changed with fresh medium for a further 24 h. All supernatants were centrifuged at 300 × *g* for 5 min to remove debris before treating HUVECs.

### 2.13. Wound Healing Assay

The HUVECs were cultured in 6-well plates with serum-free H-DMEM. When the cells filled the culture wells, cell monolayers were scratched in a straight line using a sterile pipette tip. PBS was utilized to wash cell debris. The cells were treated with supernatants derived from pretreated CFs. Twelve hours later, the images were visualized with a microscope (Nikon, Tokyo, Japan). Three fields were photographed, and the migration rate was calculated by using ImageJ software.

### 2.14. Cell Proliferation Assay

The supernatants of pretreated CFs were collected to treat HUVECs and plated in 96-well plates (3,000 cells/well). After 24, 48, and 72 h, 10% CCK-8 reagent (Beyotime Biotechnology, Shanghai, China) was added to the wells. Two hours later, the absorbance at 450 nm was obtained by using a microplate spectrophotometer (BioTek, Winooski, VT, USA).

### 2.15. Transwell Migration Assay

The HUVECs were cultured with supernatant-pretreated CFs for 24 h. HUVECs were added to the upper chambers in 200 *μ*L serum-free H-DMEM (2 × 10^4^ cells), and 600 *μ*L H-DMEM containing 10% FBS was added to the lower chambers. After 8 h at 37°C in 5% CO_2_, the migrated cells were fixed and stained with crystal violet for 10 min. The images were visualized with a microscope (OLYMPUS DP73). Three randomly selected fields were photographed, and cells were counted by using ImageJ software.

### 2.16. Tube Formation Assay

The HUVECs were cultured with supernatant-pretreated CFs for 24 h. Then, 3 × 10^4^  HUVECs/well were added to the surface of 100 *μ*L Matrigel (Corning, NY, USA) in a 96-well plate and incubated for 6 h at 37°C. Three randomly selected fields were photographed by using an inverted microscope (Nikon). Tube formation numbers and branch points were analyzed by using ImageJ software.

### 2.17. Statistical Analysis

Data were given as mean ± standard deviation. Student's *t*-test or one-way ANOVA with the post hoc test was used to compare experimental and control groups. All data were analyzed with GraphPad Prism 8.0 software. *P* < 0.05 was considered to indicate a significant difference.

## 3. Results

### 3.1. Characterization of hucMSCs, sEVs, and CFs

**Figure 1:**
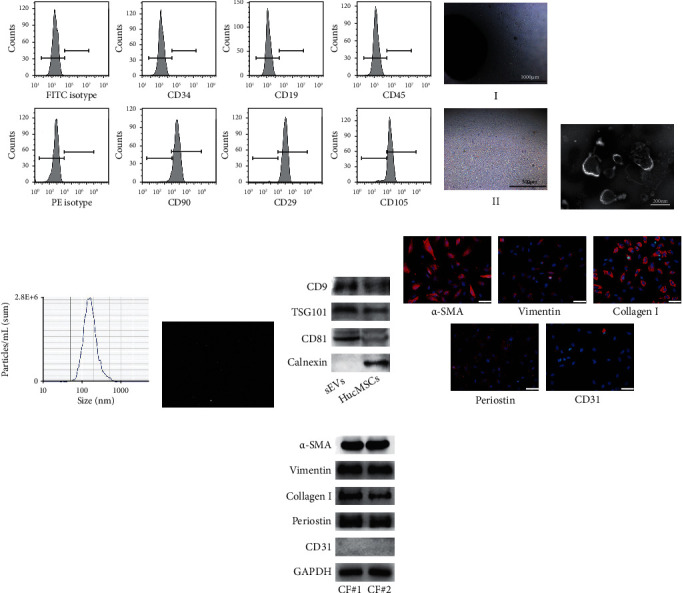
Characterization of hucMSCs, sEVs, and CFs. (a) The surface markers of hucMSCs measured by flow cytometry. (b) Morphology of hucMSCs. Scale bar: 1000 *μ*m (i) and 500 *μ*m (ii). (c) Morphology of sEVs observed by TEM. Scale bar: 200 nm. (d) Particle size distribution of sEVs measured by NTA. (e) Brownian motion image of hucMSCs. (f) Protein expression of CD9, TSG101, CD81, and calnexin in hucMSCs and sEVs. (g, h) The expression of *α*-SMA, vimentin, collagen I, periostin, and CD31 on primary CFs was measured by immunofluorescence (g) and western blotting (h). Scale bar: 50 *μ*m.

To identify the hucMSCs, flow cytometry was used to detect the surface antigenic profile of the cells. CD90, CD29, and CD105 were highly expressed in hucMSCs, but CD34, CD19, and CD45 were not expressed (Figure 1(a)). The morphology of hucMSCs was spindle-shaped (Figure 1(b)). Transmission electron microscopy (TEM) showed that the isolated vesicles had typical membrane structures (Figure 1(c)). NTA revealed that the size of sEVs was 50–160 nm (Figures 1(d) and 1(e)). To further identify the characterization of sEVs, we detected the expression of exosomal marker proteins by western blotting. CD9, TSG101, and CD81 were expressed in hucMSCs and sEVs, but calnexin was only expressed in hucMSCs (Figure 1(f)). We also measured the surface proteins of CFs by immunofluorescence and western blotting. *α*-SMA, vimentin, collagen I, and periostin were expressed in primary CFs, while CD31 was not (Figures 1(g) and 1(h)). These results provided sufficient support for subsequent experiments.

### 3.2. The sEVs Enhanced Proangiogenic Potential of CFs

**Figure 2:**
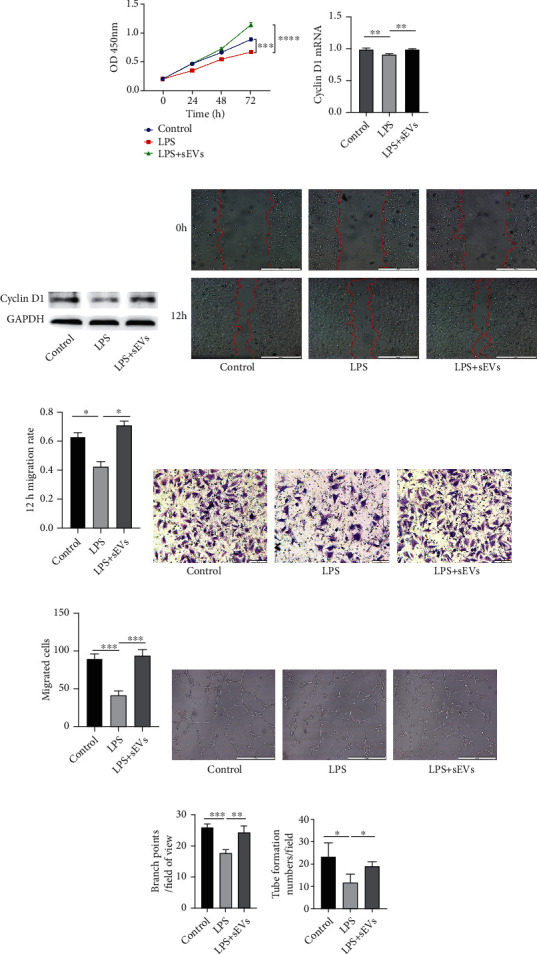
The sEVs enhanced proangiogenic potential of CFs. Primary CFs were treated with LPS (100 ng/mL) and LPS+sEVs (200 *μ*g/mL) for 24 h, and supernatants were collected. HUVECs were treated with the supernatants derived from pretreated CFs. (a) Proliferation of HUVECs was detected by the CCK-8 assay. (b) The mRNA expression of cyclin D1 was measured by qRT-PCR. (c) Cyclin D1 protein expression was detected by western blotting. (d, f) Migration of HUVECs in different treatment groups was tested by the wound healing assay (d) and Transwell migration assay (f). Scale bar: 500 *μ*m (d) and 100 *μ*m (f). (e) Quantitative analysis of 12 h migration rate in (d). (g) Quantitative analysis of migrated cells in (f). (h–j) Tube formation ability of HUVECs was measured by the tube formation assay (h). Quantitative analysis of the total branching points (i) and tube formation numbers (j). Scale bar: 500 *μ*m. ^∗^*P* < 0.05, ^∗∗^*P* < 0.01, ^∗∗∗^*P* < 0.001, and ^∗∗∗∗^*P* < 0.0001.

To investigate whether sEVs regulated CFs to promote angiogenesis in inflammatory environment, we treated primary CFs with LPS or LPS+sEVs for 24 h and then collected the supernatants. HUVECs were cultured with the supernatants for 24 h. The supernatants of CFs pretreated with sEVs promoted proliferation of HUVECs in inflammatory environment (Figure 2(a)). Additionally, the proliferation-related protein cyclin D1 was upregulated in the LPS+sEVs group compared with the LPS group (Figures 2(b) and 2(c)). The results from wound healing and Transwell assays indicated that migration of HUVECs was increased in the LPS+sEVs group compared with the LPS group (Figures 2(d)–2(g)). The tube formation assay revealed that tube formation numbers and branch points were enhanced in the LPS+sEVs group compared with the LPS group (Figures 2(h)–2(j)).

### 3.3. Angptl4 Was Increased in CFs Treated with sEVs

**Figure 3:**
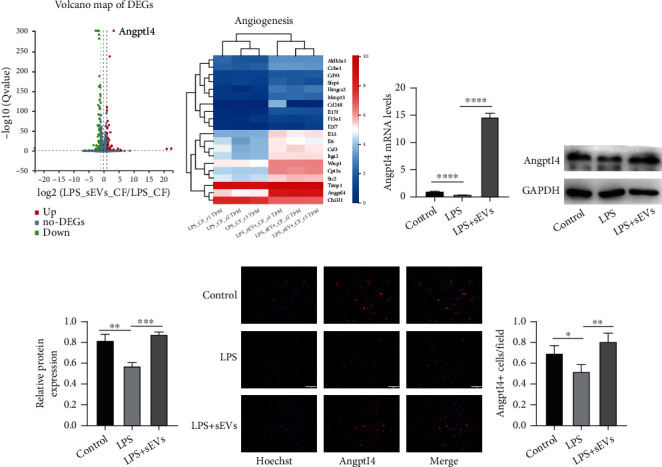
Angptl4 was increased in CFs treated with sEVs. Primary CFs were treated with LPS (100 ng/mL) and LPS+sEVs (200 *μ*g/mL) for 24 h. mRNA expression of these two groups was compared via RNA-seq analysis. (a) Volcano map of DEGs. (b) Relative fold change of the top 20 angiogenesis-related genes for enrichment analysis. (c) Angptl4 mRNA expression was viewed by qRT-PCR. (d, e) Protein expression of Angptl4 was detected by western blotting. (f, g) Immunofluorescence was used to observe Angptl4 expression. Scale bar: 100 *μ*m. ^∗^*P* < 0.05, ^∗∗^*P* < 0.01, ^∗∗∗^*P* < 0.001, and ^∗∗∗∗^*P* < 0.0001.

The sEVs enhanced proangiogenic potential of CFs. To further explore the mechanism, RNA-seq analysis of CFs was performed to find the DEGs in the LPS and LPS+sEVs groups. The volcano map of DEGs and enrichment analysis of angiogenesis-related genes showed that *Angptl4* might be an important candidate gene (Figures 3(a) and 3(b)). To verify the sequencing data, qRT-PCR suggested that sEVs increased mRNA expression of Angptl4 in CF in inflammatory environment (Figure 3(c)). Additionally, the protein level of Angptl4 was also increased in the LPS+sEVs group (Figures 3(d) and 3(e)). Immunofluorescence staining showed that Angptl4 was upregulated in the LPS+sEVs group compared with the LPS group (Figures 3(f) and 3(g)).

### 3.4. Inhibition of Angptl4 in CFs and sEVs Resulted in Decreased Angiogenesis

**Figure 4:**
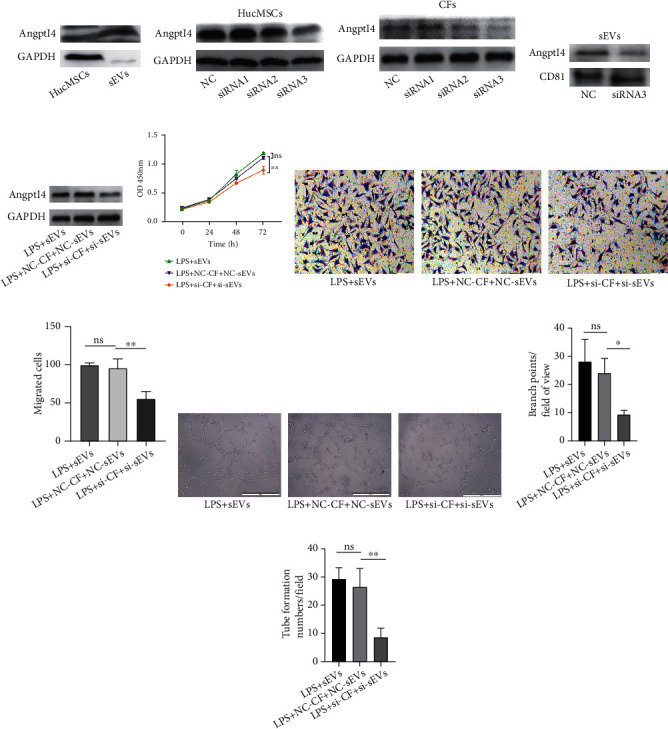
Inhibition of Angptl4 in primary CFs and sEVs resulted in decreased angiogenesis. (a) Angptl4 protein expression in hucMSCs and sEVs was detected by western blotting. (b, c) hucMSCs (b) and CFs (c) were transfected with Angptl4 siRNA, respectively, and expression of Angptl4 was measured by western blotting. (d) Angptl4 and CD81 expression in sEVs was observed by western blotting after transfection with siRNA. (e) Angptl4 expression in CFs was detected by western blotting after Angptl4 in CFs and in sEVs was knocked down with siRNA. (f) HUVECs were treated with the supernatants derived from pretreated CFs. The proliferation of HUVECs was measured by the CCK-8 assay. (g, h) The migration of HUVECs was detected by the Transwell migration assay (g). Scale bar: 100 *μ*m. Quantitative analysis of the migrated cells (h). (i–k) Tube formation ability of HUVECs was measured by tube formation assay (i). Quantitative analysis of the total branching points (j) and tube formation numbers (k). Scale bar: 500 *μ*m. ^∗^*P* < 0.05 and ^∗∗^*P* < 0.01. ns: no significant difference between two groups.

Our previous studies have demonstrated that exosomes could be taken up by CFs [11, 17]. Moreover, it was shown that in Figures 3(d) and 3(f), the protein level of Angptl4 was significantly increased in the LPS+sEVs group compared with the LPS group. The expression of Angptl4 in sEVs and hucMSCs was measured. The results showed that Angptl4 was expressed in sEVs (Figure 4(a)). There are two possible mechanisms. sEVs upregulated the expression of Angptl4 in CFs, or sEVs delivered Angptl4 to CFs. Angptl4 was knocked down in hucMSCs and CFs by siRNA. Western blotting showed that siRNA3 had the best knockdown effect in hucMSCs and CFs (Figures 4(b) and 4(c)). Angptl4 was knocked down in sEVs by an Angptl4 siRNA3 (Figure 4(d)). Moreover, compared with the NC group, Angptl4 expression in CFs was significantly decreased in the LPS+si-CF+si-sEVs group (Angptl4 was knocked down in sEVs and CFs by Angptl4 siRNA3) (Figure 4(e)). Additionally, we assessed the effect of Angptl4 on angiogenesis. The results suggested that proliferation and migration of HUVECs were decreased when Angptl4 was knocked down in sEVs and CFs (Figures 4(f)–4(h)). Furthermore, as shown in Figures 4(i)–4(k), the branch points and tube formation numbers were all reduced in the LPS+si-CF+si-sEVs group. These results indicated that Angptl4 was a key molecule in regulating CFs to promote angiogenesis.

### 3.5. Proangiogenic Potential of CFs Enhanced by sEVs Was Associated with *β*-Catenin

**Figure 5:**
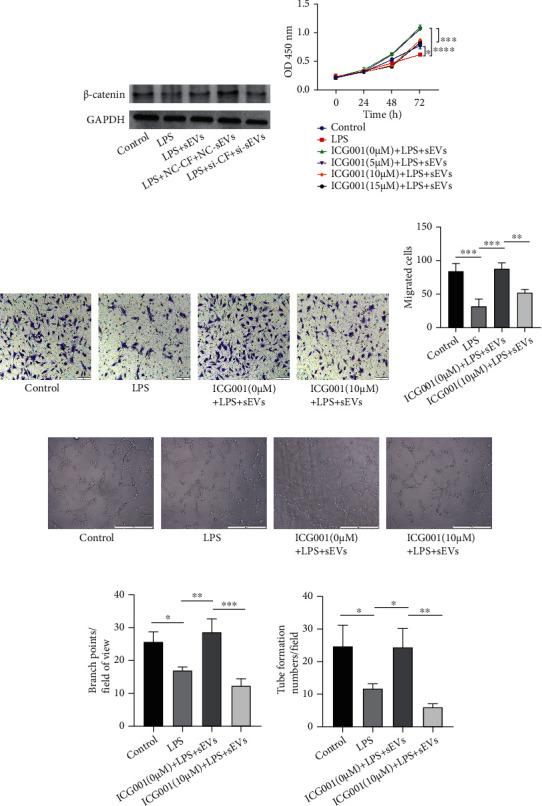
Proangiogenic potential of CFs enhanced by sEVs was associated with *β*-catenin. (a) *β*-Catenin expression in CFs was detected by western blotting after Angptl4 in CFs and in hucMSCs was knocked down with siRNA. (b) After using the *β*-catenin inhibitor ICG001 (0, 5, 10, and 15 *μ*M), primary CFs were treated with LPS (100 ng/mL) and LPS+sEVs (200 *μ*g/mL) for 24 h and supernatants were collected to culture HUVECs. The proliferation of HUVECs was measured by the CCK-8 assay. (c) The migration of HUVECs was measured by the Transwell migration assay. Scale bar: 100 *μ*m. (d) Quantitative analysis of migrated cells. (e–g) Tube formation ability of HUVECs was evaluated by the tube formation assay (e). Scale bar: 500 *μ*m. Quantitative analysis of the total branching points (f) and tube formation numbers (g). ^∗^*P* < 0.05, ^∗∗^*P* < 0.01, ^∗∗∗^*P* < 0.001, and ^∗∗∗∗^*P* < 0.0001.

The Wnt/*β*-catenin signaling pathway is involved in angiogenesis [18]. Angptl4 is also associated with *β*-catenin in skin dermal fibroblasts [14]. Although the interaction between Angptl4 and *β*-catenin has been described in other studies, the relationship in CFs has not been studied. Moreover, the regulation of Angptl4 and *β*-catenin in angiogenesis is unknown in CFs. Therefore, we detected *β*-catenin expression in CFs by western blotting. *β*-Catenin was increased in the LPS+sEVs group compared with the LPS group. After inhibiting Angptl4 expression in CFs and hucMSCs, expression of *β*-catenin was also decreased (Figure 5(a)). Therefore, Angptl4 regulated CFs to promote angiogenesis, possibly associated with *β*-catenin. To further elucidate the role of *β*-catenin in angiogenesis, CFs were treated with the *β*-catenin inhibitor ICG001. The most suitable inhibitor concentration was 10 *μ*M (Figure 5(b)). Moreover, as shown in Figure 5(b), the proliferation of HUVECs was decreased after treatment with ICG001 (10 *μ*M) compared with that in the LPS+sEVs group. The Transwell assay revealed that migration of HUVECs was also inhibited by ICG001 (Figures 5(c) and 5(d)). The tube formation assay indicated that the branch points and tube formation numbers were all reduced after treatment with ICG001 (Figures 5(e)–5(g)). These results suggested that *β*-catenin signaling was important for proangiogenic potential of CFs enhanced by sEVs.

## 4. Discussion

It has been demonstrated that exosomes derived from MSCs can promote angiogenesis after myocardial infarction, thereby improving heart function [8]. In addition, CFs as major cells have proangiogenic properties in the proliferation phase after myocardial infarction [19]. In our previous work, it was revealed that exosomes promoted fibroblast-to-myofibroblast differentiation in the inflammation phase after myocardial infarction and exert cardioprotective effects. In this study, sEVs regulated CFs to promote angiogenesis in the inflammatory environment. Angptl4 is known to be associated with angiogenesis. Combined with RNA-seq data, we found that Angptl4 was increased in CFs after sEVs treatment. Further results showed that sEVs also contained Angptl4 protein. It is possible that sEVs delivered Angptl4 to CFs or sEVs upregulated the expression of Angptl4 in CFs to promote angiogenesis. After Angptl4 was knocked down in sEVs and CFs by Angptl4 siRNA3, the angiogenic potential of CFs was decreased, which indicated that sEVs enhanced the proangiogenic potential of CFs via Angptl4. During the inflammatory stage, promoting angiogenesis may have a beneficial effect on the recovery of heart function.

It was reported that *β*-catenin was involved in angiogenesis [20]. Our findings suggested that expression of *β*-catenin in CFs was decreased after inhibiting Angptl4 expression in CFs and hucMSCs. Moreover, the proangiogenic ability of CFs enhanced by sEVs was reduced by the *β*-catenin transcription inhibitor ICG001. It has been shown that Angptl4 was expressed in the Spemann organizer of *Xenopus laevis* embryos and acted as a Wnt antagonist to promote notochord formation and prevent muscle differentiation [21]. Angptl4 binds to cadherin 11, releasing membrane-bound *β*-catenin to accelerate wound closure [14]. However, the interaction between Angptl4 and *β*-catenin has not been explained in studies of myocardial injury repair. The present study demonstrated that sEVs regulated CFs to promote angiogenesis via Angptl4. However, there were complex cell populations, including cardiomyocytes, fibroblasts, endothelial cells, and inflammatory cells in the inflammatory phase after myocardial infarction. The interactions among them and proangiogenic potential have been demonstrated [22]. Cardiomyocytes, macrophages, and fibroblasts secrete proteins, sEVs, or microvesicles, mediating intercellular communication after myocardial injury. Which cell type involves increased expression level of Angptl4, promoting angiogenesis and further improving heart function, has not been explored.

In recent years, sEVs therapy has become the focus in myocardial injury repair, although there are some challenges such as efficacy and yield issues. However, the advantages of sEVs are still promising. The sEVs drugs have gradually reached the clinical development stage and have become the next generation of potential drug delivery technology [23]. In this study, we found that sEVs regulated CFs to promote angiogenesis by increasing Angptl4 expression or delivering Angptl4 in an inflammatory environment. We elucidated the role of CFs in angiogenesis early after myocardial injury, which may provide a new idea for further research on the repair mechanism of myocardial injury and therapeutic applications of sEVs.

## 5. Conclusions

Our study demonstrated that sEVs regulated CFs to promote angiogenesis via Angptl4 in an inflammatory environment. This may provide an experimental basis for research or treatment of myocardial injury with sEVs.

## Figures and Tables

**Table 1:** Primer sequences for the amplification of target genes.

Genes	Primer sequence (5′-3′)
*β*-Actin	Forward: 5 ′-TGTCACCAACTGGGACGATA-3′
	Reverse: 5′-GGGGTGTTGAAGGTCTCA-3′

Angptl4	Forward: 5′-GCAGCCTCCTAGCCTCCTCAC-3′
	Reverse: 5′-CCACGAGACTCCAGATAGCCCTAC-3′

Cyclin D1	Forward: 5′-TTGCCCTCTGCCACAGAT-3′
	Reverse: 5′-TCAGGTTCAGGCCTTGCACT-3′

## Data Availability

The data used to support the findings of this study are available from the corresponding author upon request.
